# A machine learning model to predict critical care outcomes in patient with chest pain visiting the emergency department

**DOI:** 10.1186/s12873-021-00501-8

**Published:** 2021-10-07

**Authors:** Ting Ting Wu, Ruo Fei Zheng, Zhi Zhong Lin, Hai Rong Gong, Hong Li

**Affiliations:** 1 The School of Nursing, Fujian Medical University, Fuzhou, Fujian China; 2 Department of Emergency, Fujian Provincial Hospital, Fuzhou, Fujian China; 3 Department of Radiotherapy, Fujian Provincial Cancer Hospital, Fuzhou, Fujian China; 4 Department of Nursing, Fujian Health College, Fuzhou, Fujian China; 5 Shengli Clinical Medical College of Fujian Medical University, Fuzhou, Fujian China; 6 Department of Nursing, Fujian Provincial Hospital, Fuzhou, Fujian China

**Keywords:** Machine learning, LASSO regression, Chest pain, Critical care outcome, Prediction model, Emergency department

## Abstract

**Background:**

Currently, the risk stratification of critically ill patient with chest pain is a challenge. We aimed to use machine learning approach to predict the critical care outcomes in patients with chest pain, and simultaneously compare its performance with HEART, GRACE, and TIMI scores.

**Methods:**

This was a retrospective, case-control study in patients with acute non-traumatic chest pain who presented to the emergency department (ED) between January 2017 and December 2019. The outcomes included cardiac arrest, transfer to ICU, and death during treatment in ED. In the randomly sampled training set (70%), a LASSO regression model was developed, and presented with nomogram. The performance was measured in both training set (70% participants) and testing set (30% participants), and findings were compared with the three widely used scores.

**Results:**

We proposed a LASSO regression model incorporating mode of arrival, reperfusion therapy, Killip class, systolic BP, serum creatinine, creatine kinase-MB, and brain natriuretic peptide as independent predictors of critical care outcomes in patients with chest pain. Our model significantly outperformed the HEART, GRACE, TIMI score with AUC of 0.953 (95%CI: 0.922–0.984), 0.754 (95%CI: 0.675–0.832), 0.747 (95%CI: 0.664–0.829), 0.735 (95%CI: 0.655–0.815), respectively. Consistently, our model demonstrated better outcomes regarding the metrics of accuracy, sensitivity, specificity, positive predictive value, negative predictive value, and F1 score. Similarly, the decision curve analysis elucidated a greater net benefit of our model over the full ranges of clinical thresholds.

**Conclusion:**

We present an accurate model for predicting the critical care outcomes in patients with chest pain, and provide substantial support to its application as a decision-making tool in ED.

## Background

Globally, chest pain of acute-onset is one of the most common presenting complaints in the emergency department (ED). It represents approximately 6 to 9 million visits per year in the USA [1, 2]. In this group of patients, initial assessment is guided by vital signs, ECG findings, levels of cardiac enzymes, and estimation of established risk scores. However, this initial risk stratification remains insufficient [3], and contributes to crowding of ED and delay in patient care, ultimately resulting in greater morbidity and mortality [4]. Thus, in an overcrowded ED with limited resources, it is essential to identify critically ill patients presenting with chest pain and take appropriate measures for the preferential management of these patients [5].

In the last two decades, an evolving literature related to the identification of a wide range of critically ill patients has emerged. Previous studies have developed models for predicting the clinical deterioration of patients admitted in wards. They used mortality, cardiac arrest, and transfer to intensive care units (ICUs) as their clinical outcomes, however, they could achieve only moderate performance [6, 7]. The aim for preparing the Acute Coronary Treatment and Intervention Outcomes Network (ACTION) ICU score was to predict the complications requiring ICU care in patients with non-ST elevation myocardial infarction (NSTEMI), but reported low accuracy both in development and externally validated cohorts, thereby resulting in restricted use in clinical prediction models [8, 9]. Several established clinical outcome scores have been used for risk stratification of patients with chest pain presenting to the ED, including the History, Electrocardiography (ECG), Age, Risk factors, and Troponin (HEART) [10]; the Thrombolysis in Myocardial Infarction (TIMI) [11]; and the Global Registry of Acute Coronary Events (GRACE) score [12]. The commonly used prediction outcome is major adverse cardiovascular events (MACE) [13], namely myocardial infarction, percutaneous coronary intervention (PCI), coronary artery bypass graft (CABG), coronary artery stenosis, cardiac arrest, all-cause mortality, etc. However, there are certain differences and partial overlap in the critical care outcomes of these scores. TIMI and GRACE are time consuming and only applicable in patients with acute coronary syndrome (ACS) [14, 15], thus leading to suboptimal use in patients with chest pain of various origin. Amongst various scores, the HEART score is most accurate and widely used for risk stratification of patients with chest pain [16]. It is used for safe discharge of low-risk patients [17], or identifying high-risk patient for occurrence of MACE [18]. Considering all these facts, it can be concluded that a little or no attention is being paid to the prediction of outcomes in critically ill patients presenting with chest pain. Thus, a great challenge lies ahead in constructing a promising prediction model to identify this group of patients.

In order to improve the risk predictive ability, amongst the patients presenting to ED, various machine learning (ML) algorithms (such as support vector machine [19], neural network [20–22], random forest [21, 22], gradient-boosted decision tree [21, 22], and least absolute shrinkage and selection operator (LASSO) regression [21, 22]) have been used and demonstrated to have a satisfactory performance. Despite the advancement in the ML algorithms, major drawback of majority of these approaches is the lack of physiological sense due to the absence of a visual model [23], which may in turn result in dissatisfaction amongst and reduced use by the healthcare workers. Nevertheless, it is reported that logistic regression with LASSO regularization (LASSO regression) may address this gap. Based on the ML approach, LASSO regularization shrinks the regression coefficients toward zero, effectively selects the important predictors, and improves the interpretability of the model. With the use of this advance technology, we attempted to produce a formula, by using logistic regression, and provided a mechanistic model by nomogram.

The primary objective of this study was to develop a ML model “LASSO regression model”, using routinely available clinical features in patients with chest pain, and to accurately predict the outcomes in critically ill patients presenting at ED. Moreover, we compared the prediction performance of the LASSO regression model with the reference score.

## Methods

### Study design and setting

This was a retrospective, case-control study performed in the Fujian Provincial Emergency Center, an oldest and largest tertiary care hospital in Fujian, China. In our set-up, about 500–700 patients (including 10–12 patients with chest pain) visit the ED clinics daily, and about 50–60 patients (including 4–8 patients with chest pain) are admitted daily in the first aid room. In the present study, we included the patients with chest pain who received treatment in the first aid room. By using the National Emergency Triage Guidelines of China, with 4 levels, all patients visiting the ED were initially triaged by nurses [24]. As per the Triage Guidelines, Level 1 included the most critically ill patients that required attention in first aid room without delay. Thus, they required maximum allocation of resources, healthcare staff, and equipment for the initial management. Level 2 included critically ill patients without any danger of imminent collapse, but required to be contained in the first aid room for further examination and observation. Level 3 included patients that needs to be treated on priority in ED clinics. While, Level 4 included non-emergency patients. The study protocol was approved by the Ethics Committee Board of Fujian Provincial Hospital and the requirement of written informed consent from the study patients was waived.

### Patient population

Between January 2017 and December 2019, a total of 3146 patients with chest pain visited the ED’s first aid room and were triaged to Level 1 and 2. Patients aged 18 years or more, complaining of acute non-traumatic chest pain, and suspected to be presenting with ACS, as determined by physicians based on their clinical judgment, were included in the study. While, patients diagnosed with ACS prior to ED admission; cardiogenic chest pain such as aortic dissection, pericarditis, cardiomyopathy; non-cardiogenic chest pain caused by gastroesophageal reflux, pulmonary embolism, ruptured esophagus, tension pneumothorax, rheumatic heart diseases, cancer, etc.; and those with missing data were excluded from the study. The diagnosis of cardiogenic and non-cardiogenic chest pain was achieved according to the Chinese Expert Consensus on Standardized Evaluation and Diagnosis of Chest Pain [25]. The patients with occurrence of critical care outcomes during ED treatment were included in the Case group. While, patients without critical outcomes during ED treatment were randomly included in the Control group. In order to satisfy the assumptions of algorithm model, the number of patients in Control group were approximately equal to the Case group. The critical care outcomes were defined as either transfer to ICU, cardiac arrest, or death occurring in the ED, which were reviewed manually by scanning the electronic medical records. The patients with non-traumatic chest pain requiring ICU admission was guided by the guidelines issued by the Emergency Medicine Branch of the Chinese Medical Association. As per these guidelines, the patients with non-traumatic chest pain were admitted to the ICU, if they fulfilled at least one of the following seven criteria [26]: altered consciousness, arterial oxygen saturation < 90% or respiratory failure, significant abnormalities in blood pressure (BP), hemodynamics affected by severe arrhythmia, previous Marfan’s syndrome with severe high BP, and breathing difficulties or full chest on the affected side. The cardiac arrest was defined by unresponsiveness, apnea, and the absence of a central palpable pulse due to pulseless ventricular tachycardia (PVT), ventricular fibrillation (VF), pulseless electrical activity (PEA), or asystole [27]. Data prior to cardiac arrest was used in patients who suffered both cardiac arrest and required ICU transfer, or both cardiac arrest and subsequent death during the treatment in ED.

### Patient characteristics and data collection

The routinely available information in the ED was used for the critical prediction model. The information included; 1) Demographics details: age, gender, mode of arrival (ambulance use, transfer from other hospital, walk-in, intra-hospital transfer, and others), and admission and discharge time; 2) Risk factors: history of tobacco use in any form, and family history of premature coronary artery disease (CAD), diabetes, hypertension, hyperlipidemias, and obesity; 3) ED presentation: quality, location, and duration of chest pain, time of arrival, height, weight, Killip class, vital signs and mental status at triage, and complications such as acute heart failure; 4) Initial evaluation: ECG findings (including characteristics of ECG, QT interval, QTc interval, the change of ST-segment, non-specific abnormalities, etc.) and laboratory tests [N-terminal pro brain natriuretic peptide (NT-proBNP), cardiac troponin I (cTnI), serum creatinine (SCr), creatine kinase (CK), and creatine kinase-MB (CKMB)]; 5) Medical treatment: current reperfusion therapy [PCI, CABG, or none]. To ensure the quality of the data, the clinical information and outcomes of all the patients were extracted manually from the medical records. Two researchers were independently involved in data collection. While one screened the participants from electronic medical records based on the inclusion and exclusion criteria, another blinded to clinical outcomes, reviewed all available ED records for complete assessment.

### Model development and validation

The data was randomly divided into two sets, the training set (70% of the patients) and the testing set (30% of the patients). In training set, LASSO regression was used to effectively select the important predictors and improve the interpretability of the model through shrink regression coefficients toward zero. Multivariate logistic regression analysis was used to generate independent predictors of critical care outcome in patients with chest pain. Finally, based on nomogram, a visualized LASSO prediction model was established. In both the sets, we computed the model performance, as the discrimination, and calculated 1) Area under the receiver-operating-characteristics curve (AUC); 2) Results of confusion matrix (i.e., accuracy, sensitivity, specificity, positive predictive value (PPV), negative predictive value (NPV), and F1 score); and 3) Net benefit through decision curve analysis. The calibration was appraised by the Hosmer-Lemeshow (HL) test. Moreover, to evaluate the superiority of prediction capability of LASSO model, based on the above metrics, we compared it with the reference model i.e., HEART, GRACE, and TIMI score.

### Statistical analysis

Normality of the data was test by Kolmogorov-Smirnov test. If the continuous variable were normally distributed, then they were represented as mean ± standard deviation (SD), else they were represented as median [interquartile range (IQR)]. While, categorical variables were presented as frequencies (percentages). Between group comparison of categorical and continuous data was performed by the Student’s t-test or Wilcoxon’s test, and Chi-square or Fisher’s test, respectively. Missing values were imputed by random forest. All statistical analyses were performed with R software (version 3.5.1; http://www.Rproject.org). A *P* value less than 0.05 was regarded as statistically significant.

## Results

### Patient characteristics

**Fig. 1:**
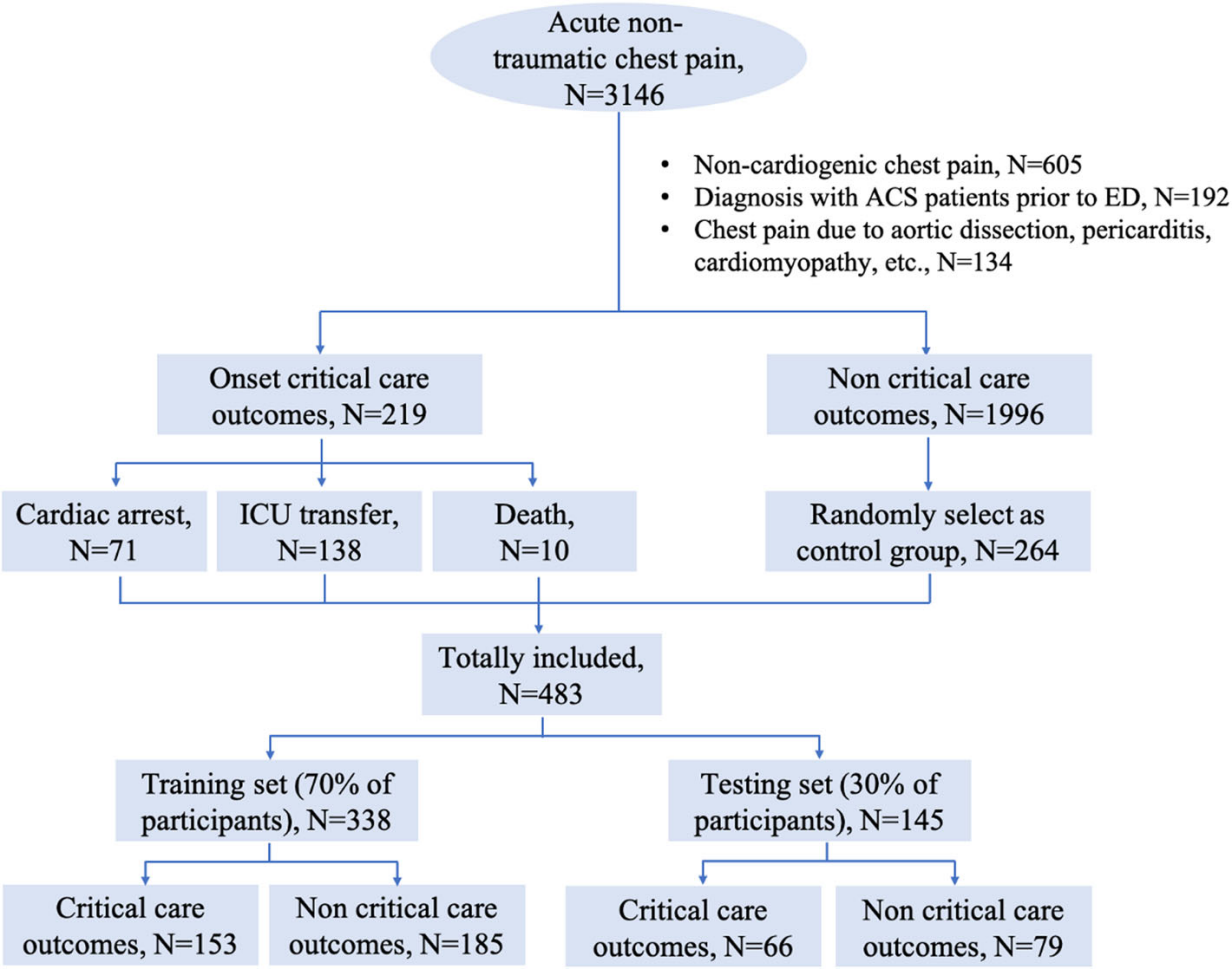
Flowchart of the patient recruitment

The study consisted of 219 patients with critical care outcomes in the Case group, and randomly selected 264 stable patients in the Control group. Then, we randomly assigned 338 (70%) patients to the training set, and the remaining 145 (30%) patients to the testing set, as illustrated in Fig. 1. Patient characteristics in the training and testing sets are depicted in Table 1. There was no significant difference between the two sets in terms of any of the characteristics evaluated.

**Table 1:** Characteristics of Training ang Testing sets

Features	Training set ***N*** = 338, N(%)/M (IQR)	Testing set ***N*** = 145, N(%)/M (IQR)	***Z/χ***^***2***^	***P*** values
**Age, years**^**b**^	64 (53–73)	66 (53–74.5)	0.984	0.352
**Critical care outcomes**	153 (45.3)	66 (45.5)	< 0.001	0.959
**Male**	261 (77.2)	112 (77.2)	< 0.001	0.996
**Mode of arrival**				
Ambulance use	22 (6.5)	9 (6.2)	3.37	0.498
Transfer from other hospital	44 (13.0)	24 (16.5)		
Walk in	268 (79.3)	108 (74.5)		
Intra-hospital transfer	2 (0.6)	1 (0.7)		
Others	2 (0.6)	3 (2.1)		
**History**^**a**^	9 (2.7)	1 (0.7)	1.948	0.163
**Diabetes**	94 (27.8)	37 (25.5)	0.27	0.603
**Hypertension**	193 (57.1)	86 (59.3)	0.203	0.652
**Dyslipidemia**	103 (30.5)	45 (31.0)	0.015	0.902
**Smoking**	144 (42.6)	55 (37.9)	0.914	0.339
**Obesity**^**a**^	4 (1.2)	2 (1.4)	0.032	0.859
**Number of risk factors**^**a**^				
0	47 (13.9)	25 (17.2)	1.58	0.812
1	110 (32.5)	49 (33.8)		
2	119 (35.2)	44 (30.3)		
3	54 (16.0)	23 (15.9)		
4	8 (2.4)	4 (2.8)		
**Acute heart failure**	37 (10.9)	11 (7.6)	1.28	0.258
**Type of chest pain**				
Only atypical symptoms	39 (11.5)	17 (11.7)	0.429	0.807
Typical and atypical symptoms	100 (29.6)	47 (32.4)		
Only typical symptoms	199 (58.9)	81 (55.9)		
**Duration of chest pain**^**b**^				
<24 h	209 (61.8)	94 (64.8)	0.718	0.698
24 h-7d	80 (23.7)	34 (23.4)		
>7d	49 (14.5)	17 (11.7)		
**Electrocardiogram findings**				
Normal	68 (20.1)	28 (19.3)	0.048	0.976
Nonspecific abnormalities	135 (39.9)	58 (40.0)		
Ischemia	135 (39.9)	59 (40.7)		
**Killip class**				
I	203 (60.1)	93 (64.1)	0.983	0.805
II	68 (20.1)	24 (16.6)		
III	23 (6.8)	10 (6.9)		
IV	44 (13.0)	18 (12.4)		
**Reperfusion therapy**^**b**^				
PCI	90 (26.6)	49 (33.8)	2.585	0.275
CABG	2 (0.6)	1 (0.7)		
None	246 (72.8)	95 (65.5)		
**Temperature**^**a**^, °C				
36.0 ≤ T ≤ 38.0	324 (95.9)	143 (98.6)	2.418	0.12
< 36.0 or > 38.0	14 (4.1)	2 (1.4)		
**Heart rate**, beats/min				
60–100	232 (68.6)	114 (78.6)	4.976	0.026
< 60 or > 100	106 (31.4)	31 (21.4)		
**Respiratory rate,** beats/min				
11–20	196 (58.0)	81 (55.9)	0.188	0.665
≤ 10 or > 20	142 (42.0)	64 (44.1)		
**Systolic pressure**^**b**^, mmHg				
≥ 90	308 (91.1)	137 (94.5)	1.579	0.209
< 90	30 (8.9)	8 (5.5)		
**Diastolic pressure**, mmHg				
≥ 60	286 (84.6)	130 (89.7)	2.157	0.142
< 60	52 (15.4)	15 (10.3)		
**Conscious state**^**b**^				
Alert	336 (99.4)	143 (98.6)	0.766	0.381
**SCr** ^**b**^, μmol/L				
< 186	322 (95.3)	138 (95.2)	1.383	0.501
186–451	11 (3.3)	3 (2.1)		
>451	5 (1.5)	4 (2.8)		
**cTnI**, μg/L				
< 0.2	82 (24.3)	35 (24.1)	3.753	0.153
0.2–10.0	225 (66.6)	104 (71.7)		
> 10.0	31 (9.2)	6 (4.1)		
**CKMB**^**b**^**,** U/L	28.5 (11 ~ 101.5)	22 (12 ~ 59)	−0.894	0.371
**NT-proBNP**^**b**^, ng/L	1539.69 (485.25 ~ 3618.75)	1539.69 (305.85 ~ 3349)	−0.809	0.419
**CK**^**b**^, U/L	310 (95 ~ 910.5)	190 (78.5 ~ 491)	−2.186	0.029

*PCI* percutaneous transluminal coronary intervention, *CABG* coronary artery bypass grafting, *SCr* serum creatinine, *cTnI* cardiac troponin I, *CKMB* creatine kinase-MB, *NT-proBNP* N-terminal pro brain natriuretic peptide, *CK* creatine kinase

^a^Fisher’s test

^b^Wilcoxon’s test

### Selection of features for critical care outcomes in patients with chest pain

**Fig. 2:**
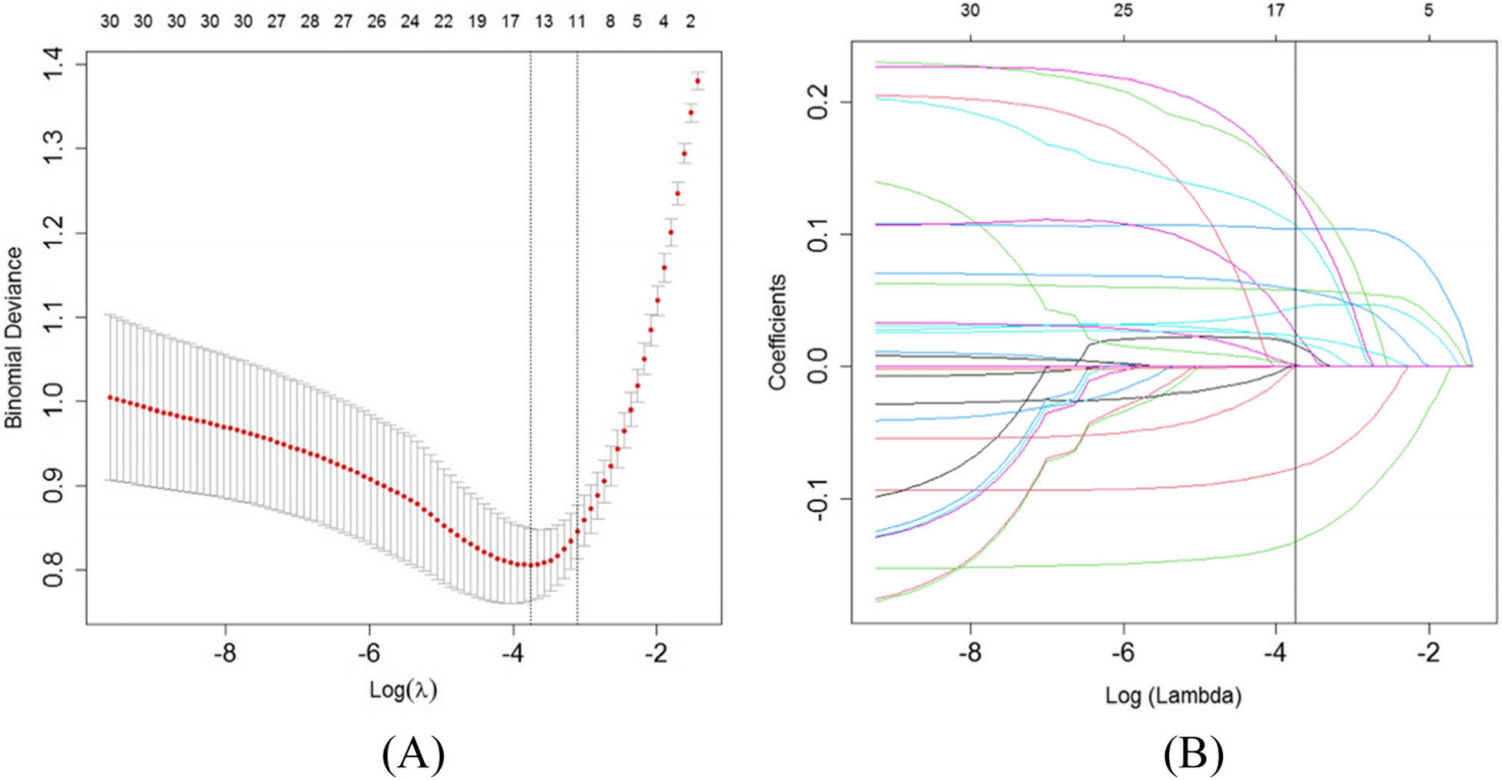
Critical care outcomes risk factors selection using the least absolute shrinkage (**A**) and selection operator (LASSO) binary logistic regression model (**B**)

On the basis of 338 patients in the training set, 40 features were reduced to 14 potential predictors and these features had non-zero coefficients in the LASSO regression model, illustrated in Fig. 2. These features included gender, mode of arrival, smoking, number of risk factors, reperfusion therapy, Killip class, ECG findings, temperature, respiratory rate, systolic BP (SBP), shock index, SCr, CKMB, and BNP.

### Development of a critical care outcome prediction model

**Fig. 3:**
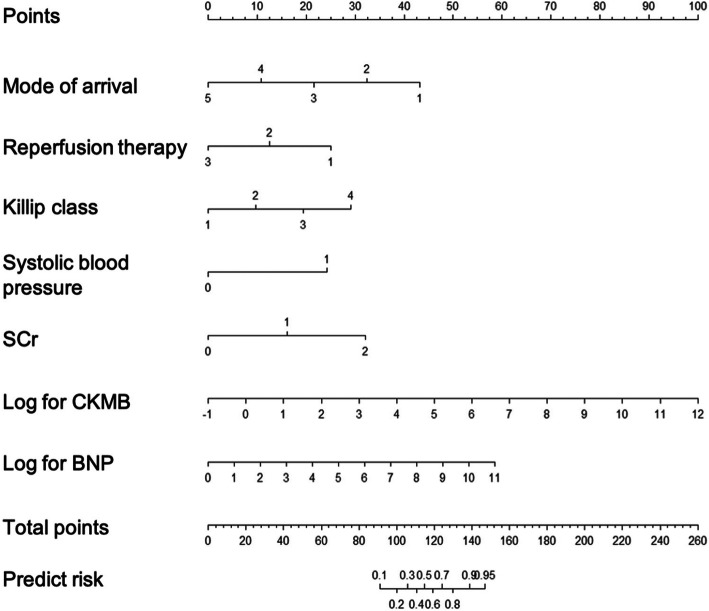
Nomogram for prediction of critical care outcomes in patients with chest pain. SCr: serum creatinine, CKMB: creatine kinase-MB, BNP: brain natriuretic peptide

Multivariable logistic regression analysis identified model of arrival, reperfusion therapy, Killip class, SBP, SCr, CKMB, and BNP as independent predictors of critical care outcomes in patients with chest pain (Table 2). These seven independent predictors were then used to develop a LASSO regression model, which was presented as a nomogram (Fig. 3). Further, we developed a point score for predicting the outcomes in critically ill patients, as depicted in Table 3. The probability and risk stratification for each score point is depicted in Table 4.

**Table 2:** Risk factors for critical care outcome among chest pain patients

Risk factors	β	S.E	***Z***	***P*** value	***OR (95%CI)***
**Mode of arrival**	− 0.995	0.305	−3.265	0.001	0.37 (0.204–0.672)
**Reperfusion therapy**	−1.151	0.193	−5.970	0.000	0.316 (0.217–0.461)
**Killip**	0.893	0.190	4.702	0.000	2.443 (1.684–3.545)
**SBP**	2.228	0.692	3.218	0.001	9.281 (2.389–36.05)
**SCr**	1.480	0.632	2.343	0.019	4.395 (1.274–15.161)
**CKMB**	0.709	0.130	5.460	0.000	2.031 (1.575–2.62)
**NT-proBNP**	0.490	0.135	3.626	0.000	1.633 (1.253–2.129)

*SBP* systolic blood pressure, *SCr* serum creatinine, *CKMB* creatine kinase-MB, *NT-proBNP* N-terminal pro brain natriuretic peptide

**Table 3:** Risk score for critical care outcome among chest pain patients

Risk factors	Scores	Risk factors	Scores
**Mode of arrival**		**Killip**	
Ambulance use	43	I	0
Transfer from other hospital	32	II	10
Walk in	22	III	19
Intra-hospital transfer	11	**IV**	29
Others	0		
**Reperfusion therapy**		**SCr**	
PCI	25	< 186	0
CABG	12	186–451	16
None	0	>451	32
**CKMB**		**NT-proBNP**	
0.37	0	1.00	0
1.00	8	2.72	5
2.72	15	7.39	11
7.39	23	20.09	16
20.09	31	54.60	21
54.60	38	148.41	27
148.41	46	403.43	32
403.43	54	1096.63	37
1096.63	62	2980.96	43
2980.96	69	8103.08	48
8103.08	77	22,026.47	53
22,026.47	85	59,874.14	59
59,874.14	92		
162,754.79	100		

*PCI* percutaneous transluminal coronary intervention, *CABG* coronary artery bypass grafting, *SCr* serum creatinine, *CKMB* creatine kinase-MB, *NT-proBNP* N-terminal pro brain natriuretic peptide

**Table 4:** Probability of critical care outcome and corresponding risk stratification

Scores	Predicted Risk	Risk group
91	0.1	low risk
100	0.2	low risk
106	0.3	low risk
111	0.4	intermediate risk
115	0.5	intermediate risk
119	0.6	intermediate risk
124	0.7	high-risk
130	0.8	high-risk
139	0.9	high-risk

### Performance of the critical care outcomes prediction model

**Fig. 4:**
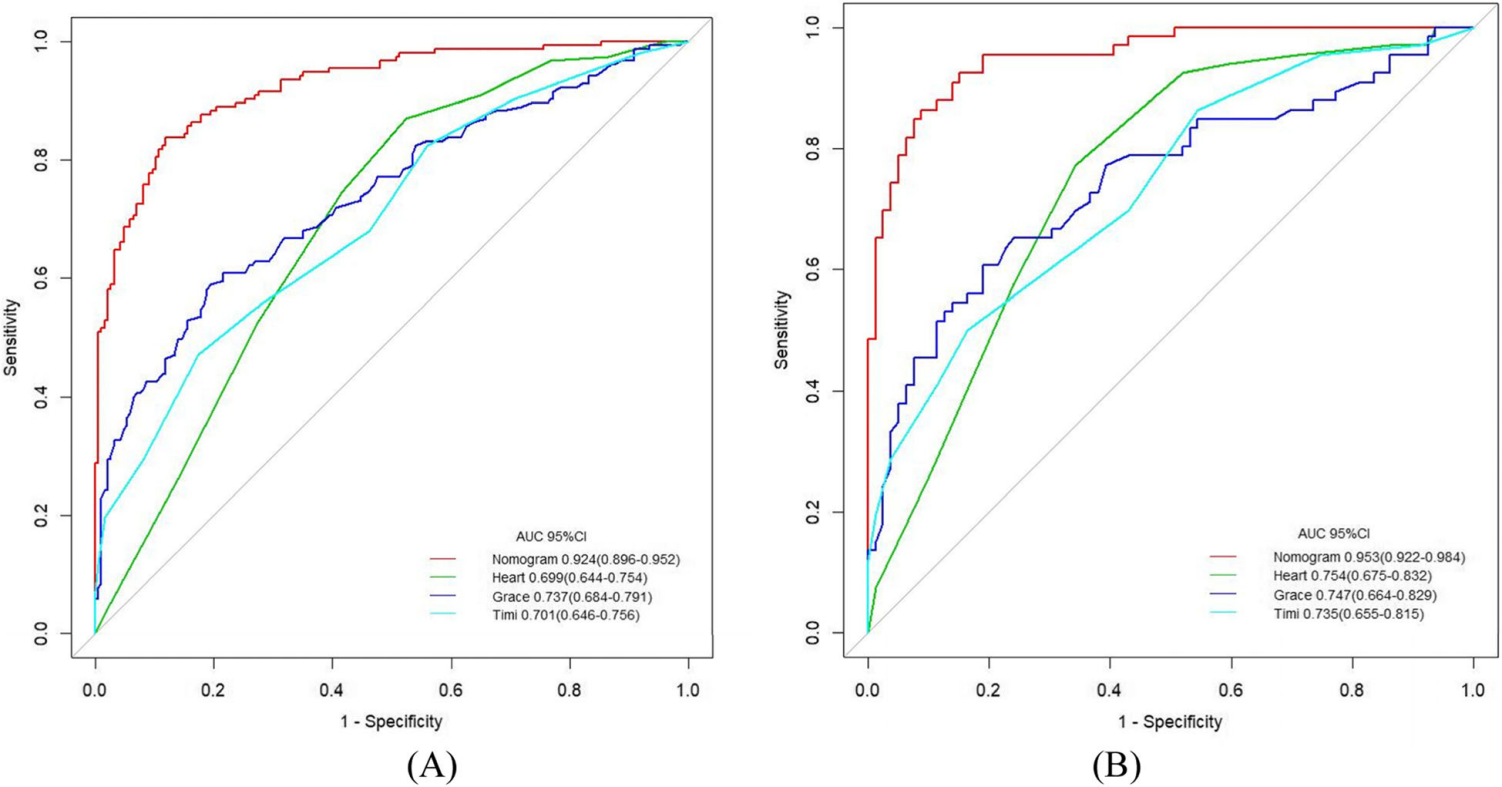
ROC for the critical care outcomes prediction in training set (**A**) and testing set (**B**)

**Fig. 5:**
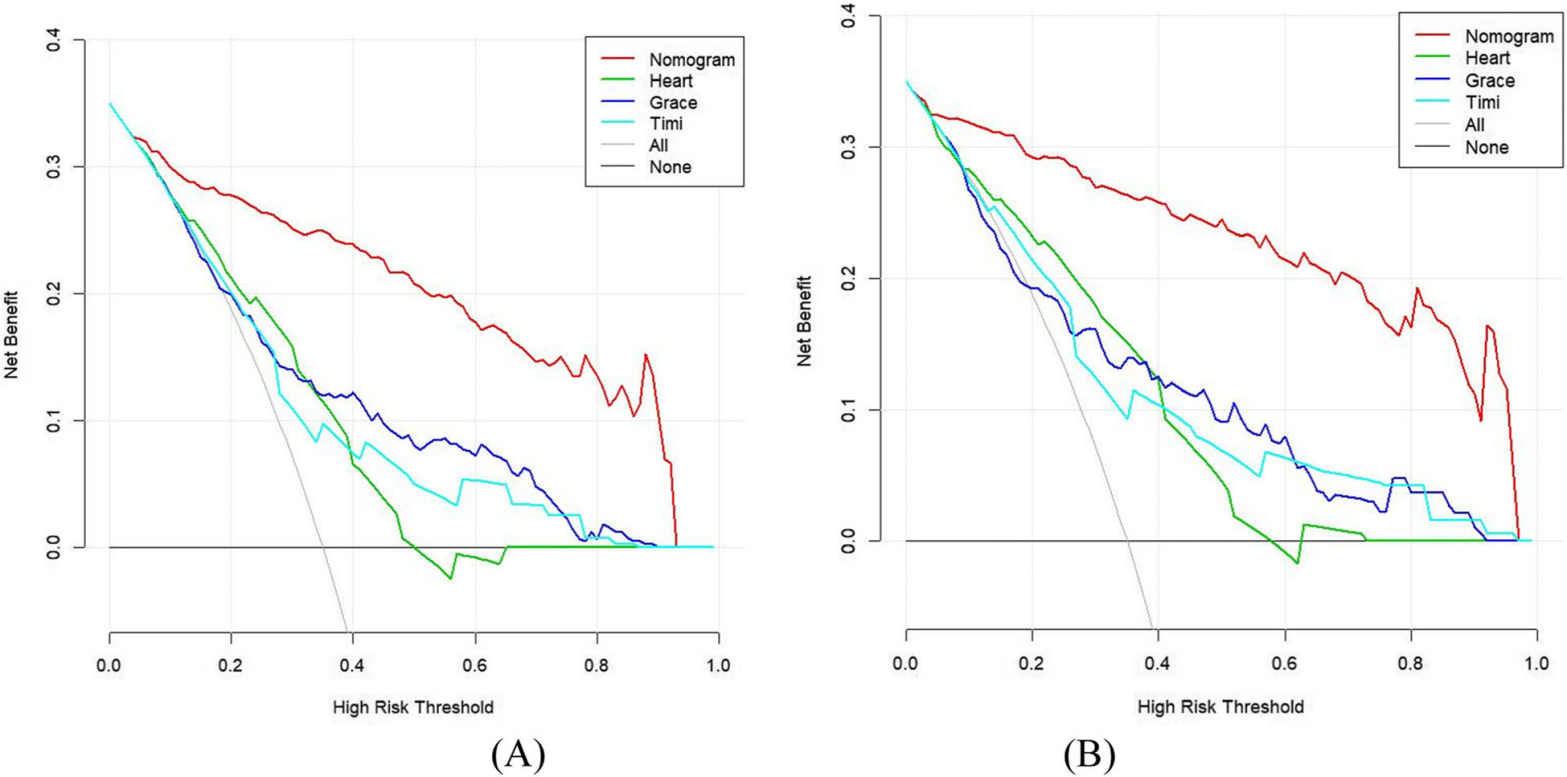
Decision curve analysis for the critical care outcomes prediction in training set (**A**) and testing set (**B**)

For the discrimination based on the training set, LASSO regression model achieved a good result with AUC of 0.924 (95%CI: 0.896–0.952), which was superior to HEART, GRACE, and TIMI score with AUC of 0.699 (95%CI: 0.644–0.754), 0.737 (95%CI: 0.684–0.791), and 0.701 (95%CI: 0.646–0.756), respectively, as illustrated in Fig. 4A. Moreover, compared with these three reference models, LASSO regression model demonstrated a higher accuracy, sensitivity, specificity, PPV, NPV, and F1 score, as depicted in Table 5. With regard to the calibration, the HL test yielded non-significant statistic in LASSO regression model (*P* value = 0.983), which suggested that there was no departure from perfect fit. Similarly, the decision curve analysis (illustrated in Fig. 5A) demonstrated that the net benefit of LASSO regression model surpassed that of the comparison models throughout the threshold range.

**Table 5:** Performance of LASSO and comparison models in predicting the critical care outcomes in patients with chest pain

	Accuracy	Sensitivity	Specificity	PPV/Precision	NPV	F1	Cut-off	AUC	95%CI
**Training set**									
LASSO	0.861	0.847	0.881	0.853	0.867	0.845	113.0	0.924	0.896–0.952
HEART	0.654	0.839	0.476	0.578	0.815	0.694	5.5	0.699	0.644–0.754
GRACE	0.707	0.588	0.805	0.714	0.703	0.645	145.5	0.737	0.684–0.791
TIMI	0.665	0.471	0.826	0.692	0.652	0.560	5.5	0.701	0.646–0.756
**Testing set**									
LASSO	0.890	0.864	0.911	0.891	0.889	0.877	117.0	0.953	0.922–0.984
HEART	0.710	0.773	0.658	0.654	0.776	0.708	6.5	0.754	0.675–0.832
GRACE	0.717	0.606	0.810	0.727	0.711	0.661	141.5	0.747	0.664–0.829
TIMI	0.683	0.500	0.835	0.717	0.667	0.589	4.5	0.735	0.655–0.815

*PPV* positive predictive value, *NPV* negative predictive value, *AUC* area under the receiver-operating-characteristics curve

### Validation of the critical care outcomes prediction model

For the discrimination based on the testing set, LASSO regression model with AUC of 0.953 (95%CI: 0.922–0.984) outperformed the reference models HEART, GRACE, and TIMI score with AUC of 0.754 (95%CI: 0.675–0.832), 0.747 (95%CI: 0.664–0.829), and 0.735 (95%CI: 0.655–0.815), respectively, illustrated in Fig. 4B. Consistently, LASSO regression model produced better outcomes regarding the metrics of accuracy, sensitivity, specificity, PPV, NPV, and F1 score, as depicted in Table 5. Good calibration was observed for the probability of critical care outcomes, with HL test reporting a non-significant statistic for our model (*P* value = 0.854 and 0.737, respectively). Most importantly, the decision curve analysis demonstrated that LASSO regression model has a higher value on clinical application than HEART, GRACE, and TIMI score, as illustrated in Fig. 5B.

## Discussion

We applied machine learning approach - LASSO regression model - to predict the likelihood of complications requiring ICU care, cardiac arrest, or mortality amongst the patients with chest pain admitted in ED. The LASSO regression model comprises of 7 clinical features available at initial contact of patient with ED i.e., mode of arrival, reperfusion therapy, Killip class, SBP, SCr, CKMB, and BNP. Compared to the reference models, the LASSO regression model demonstrated a superior performance in predicting critical care outcomes, including improved AUC value and other metrics. Moreover, the decision curve analysis revealed that LASSO regression model yields a larger net benefit—the trade-off between appropriate prediction and over-prediction—throughout the full range of thresholds. The use of this objective risk stratification tool may help the hospitals effectively use the limited ED resources while ensuring that high-risk chest pain patients are taken care of safely.

The reasons for the improved predictive abilities observed with the LASSO regression model are multifactorial. Firstly, the present study employed complete set of information; for instance, demographics, risk factors of ACS, ED presentation, initial laboratory and ECG findings, and medical treatment, all of these could have resulted in an improved predictive ability. Raita et al. [22] proposed an ED triage system to predict critical care outcomes—direct admission to an ICU or in-hospital death—based on the limited set of predictors only collected at ED triage, such as demography, triage vital signs, chief complaints, and patient comorbidities. Compared with this ED triage systems, the LASSO regression model outperformed in terms of the value of AUC, sensitivity, and specificity (0.86, 0.80, and 0.76, respectively). This is attributable, at least in part, to the limited set of predictors employed. Nevertheless, the differential study purposes and participants make it impossible to compare both the systems. The risk score generated by Raita et al. was proposed for emergency triage and rapid identification of the priority patients. However, it could compromise the accuracy. Contrarily, the LASSO regression model is proposed to be used in emergency room rather than at triage. We ensured the patient safety first, even though time consuming. The participants enrolled in both the studies make it impossible to compare both the systems, and the risk score proposed for emergency patients cannot be directly generalized to patients with chest pain. Moreover, the risk score, in the present study, had some indicators that overlapped with ACTION ICU score [8], which included SCr, SBP, and reperfusion therapy. The findings of some previous studies are inconsistent with that of the present study, notably, cTnI and CKMB have been used for several decades in diagnosing patients with chest pain and to stratify them into those with myocardial and non-myocardial infarction [28]. Estimation of serum troponin is the golden standard of evaluating the cardiac markers [26], however in the present study, cTnI failed to show a statistically significant difference in recognizing the critical care outcomes, nevertheless serum CKMB was measured. A plausible explanation for this inconsistency may be due to the differences in testing point between these two cardiac markers, where cTnI was estimated at an emergency triage immediately on arrival at the ED, and the CKMB was measured before the occurrence of critical care outcomes during the emergency room treatment. Thus, there was an obvious time difference in their estimation. Moreover, in ACTION ICU score, prior revascularization was associated with lower likelihood of developing in-hospital complications requiring ICU care. While, conversely, in the present study, it served as a risk factor because reperfusion therapy was defined as a current therapy, and may have a higher likelihood of presenting to the ICU post-operation.

Secondly, an alternative approach to enhance the predictive ability is to utilize advanced ML algorithm, which is capable of handling high-order interactions amongst the enormous predictors, remarkably, combing them in non-linear highly interactive ways [29]. Recently, ML approaches have opened up vast possibilities in emergency medicine—e.g., cardiac complications in patients with acute chest pain [30], cardiac arrest in ED patients [19, 20], and an ED triage tool for all adults patients [22] or children [21]. The present study confirms that ML models can attain a superior predictive ability for critical care outcomes in patients with acute chest pain. While over-fitting, often generates spurious correlations in the data, we were seriously concerned and thus, adopted multiple rigorous approaches to mitigate, regularize, and validate the independent cohort. Consequently, the performance of the validation model exceeded that of the development model. Decision curve analysis was used to evaluate the feasibility of the proposed model, the result demonstrated that we need to have an optimum balance between under-prediction and over-prediction. The ML model, used in the present study, enables correct identification of the critically ill patients, which might be inappropriately under-triaged into the lower-risk by the HEART, GRACE, TIMI score. Similarly, our model could rule out stable patient which would be over-triaged into high risk patient with those three reference scores, and thus may require additional resources. This finding supports the generalizability of the LASSO regression model. Moreover, this model can be employed directly during the bedside rounds.

Finally, due to the wide usage of HEART, GRACE, and TIMI scores and specifically recognized ability to stratify the patients with cardiovascular system, it may be the best reference models for the present study. We found that our LASSO regression model outperformed to these three well-known scores, and all of these showed a low discrimination index (AUC). Moreover, HEART score was slightly higher than the other two clinical risk scores in detecting the critically ill patients with chest pain in the testing set. To our knowledge, this is the first study to use HEART, GRACE, and TIMI scores in predicting critical care outcomes with the patient of chest pain. We speculate that the poor performance of these three risk scores might be attributed to the indicator of ECG test result they included, such as ST-elevation or ST-depression. There is a time window of clinical deterioration in patients with chest pain. Riley et al. found that 11% of patients with ST-elevation myocardial infarction (STEMI) had an initial non-diagnostic ECG with median time of 72 min between symptom onset and first medical contact, while 72.4% of these patients had an elevation of ST segment after 90 min [31]. Likewise, in the present study, these three scores was calculated only by applying the clinical data at triage, where some predictors remained normal. Several studies [15, 16, 18, 32] have previously compared the prognostic value of different risk scores for predicting MACE among chest pain patients, which consistently favored HEART score over other clinical risk scores in stratifying high-risk patients regarding the onset of MACE. However, in the present study, this prominence was not obviously evident due to the heterogeneity of the study populations, because more severe patients (high risk patients) with Level 1 and Level 2 chest pain who received treatment in the first aid room were included. A meta-analysis demonstrated that the discrimination accuracy of HEART score for the low-risk group was significantly higher than that of the high-risk group [17]. Irrespective of cause, our ML model resulted in an outstanding performance in terms of the AUC value, results of confusion matrix, and the assessment of clinical use.

The current study had several potential limitations. Firstly, this was a single-center study performed at a tertiary provincial emergency center in China, thus the institutional factors and potential selection bias might have resulted in findings that are less generalizable. Secondly, it is a fundamental case-control study of medical history data, with an intrinsic limitation in precision that entails. Nevertheless, most of the events contemplated are concrete and were truly recorded in the electronic medical records system. Thirdly, though the proposed model has demonstrated perfect performance in internal validity, there is a need for external validation of the scores for routine clinical use. Fourthly, we did not capture other clinical features, such as heart rate variability (HRV). The HRV has been regarded as a promising predictor that is recognized to have a significant relationship between the autonomic nervous system and cardiovascular mortality [30, 33–35]. Due to the complicated estimation, time consuming procedure, and unsuitability with non-sinus rhythm [35], HRV has not been widely used clinically, especially in the developing country, and thus, was not included in the present study. Finally, the indication and clinical threshold of ICU admission vary depending on the local healthcare resource, such as ICU transfer criteria, ICU bed availability, and the ratio of nurse/patient and nurse/doctor.

## Conclusions

To conclude, based on the ML model, we proposed a visualized LASSO regression model using 7 routinely captured clinical features. Compared to well-known clinical risk score—HEART, GRACE and TIMI score, our model had a superior performance in predicting the critical care outcomes in patients with chest pain. Moreover, the model minimized the potential over-predicted and under-predicted critical care outcomes that could result in excessive resource allocation to low-risk patients and insufficient treatment of high-risk patients. While external validation remains essential, the present study may pave the way for the application of ML-based predication models in critically ill patient with chest pain, as a decision-making technological tool.

## Data Availability

The datasets used and/or analyzed during the current study are available from the corresponding author on reasonable request.

## Abbreviations

AUC: Area under the receiver-operating-characteristics curve
BP: Blood pressure
CK: Creatine kinase
CKMB: Creatine kinase-MB
cTnI: Cardiac troponin I
ED: Emergency department
GRACE: The Global Registry of Acute Coronary Events
HEART: History, Electrocardiography, Age, Risk factors, and Troponin
LASSO: Least absolute shrinkage and selection operator
ML: Machine learning
NT-proBNP: N-terminal pro brain natriuretic peptide
NPV: Negative predictive value
PPV: Positive predictive value
SCr: Serum creatinine
TIMI: The Thrombolysis in Myocardial Infarction

## References

[1] Virani SS, Alonso A, Benjamin EJ, Bittencourt MS, Callaway CW, Carson AP, Chamberlain AM, Chang AR, Cheng S, Delling FN, Djousse L, Elkind MSV, Ferguson JF, Fornage M, Khan SS, Kissela BM, Knutson KL, Kwan TW, Lackland DT, Lewis TT, Lichtman JH, Longenecker CT, Loop MS, Lutsey PL, Martin SS, Matsushita K, Moran AE, Mussolino ME, Perak AM, Rosamond WD, Roth GA, Sampson UKA, Satou GM, Schroeder EB, Shah SH, Shay CM, Spartano NL, Stokes A, Tirschwell DL, VanWagner L, Tsao CW, American Heart Association Council on Epidemiology and Prevention Statistics Committee and Stroke Statistics Subcommittee (2020). Heart disease and stroke Statistics-2020 update: a report from the American Heart Association. Circulation..

[2] NHAMC S. 2015 Emergency department summary tables. CDC. Washington. http://www.cdc.gov/nchs/data/ahcd/nhamcs_emergency/2010_ed_web_tables.pdf.2015.

[3] Thang ND, Sundstrom BW, Karlsson T (2014). ECG signs of acute myocardial ischemia in the prehospital setting of a suspected acute coronary syndrome and its association with outcomes. Am J Emerg Med.

[4] Sun BC, Hsia RY, Weiss RE, Zingmond D, Liang LJ, Han W, McCreath H, Asch SM (2013). Effect of emergency department crowding on outcomes of admitted patients. Ann Emerg Med.

[5] Nannan Panday RS, Minderhoud TC, Alam N, Nanayakkara PWB (2017). Prognostic value of early warning scores in the emergency department (ED) and acute medical unit (AMU): a narrative review. Eur J Intern Med.

[6] Churpek MM, Yuen TC, Edelson DP (2013). Predicting clinical deterioration in the hospital: the impact of outcome selection. Resuscitation..

[7] Town JA, Churpek MM, Yuen TC, Huber MT, Kress JP, Edelson DP (2014). Relationship between ICU bed availability, ICU readmission, and cardiac arrest in the general wards. Crit Care Med.

[8] Guimaraes PO, Sampaio MC, Malafaia FL (2020). Clinical outcomes and need for intensive care after non-ST-segment-elevation myocardial infarction. Eur J Intern Med.

[9] Fanaroff AC, Chen AY, Thomas LE, et al. Risk Score to Predict Need for Intensive Care in Initially Hemodynamically Stable Adults With Non-ST-Segment-Elevation Myocardial Infarction. J Am Heart Assoc. 2018;7(11):e008894. 10.1161/JAHA.118.008894 PMC6015341 29802146

[10] Six AJ, Cullen L, Backus BE, Greenslade J, Parsonage W, Aldous S, Doevendans PA, Than M (2013). The HEART score for the assessment of patients with chest pain in the emergency department: a multinational validation study. Crit Pathw Cardiol.

[11] Antman EM, Cohen M, Bernink PJ (2000). The TIMI risk score for unstable angina/non-ST elevation MI: a method for prognostication and therapeutic decision making. JAMA..

[12] GRACE Investigators. Rationale and design of the GRACE (Global Registry of Acute Coronary Events) Project: a multinational registry of patients hospitalized with acute coronary syndromes. Am Heart J. 2001;141(2):190–9. 10.1067/mhj.2001.112404 11174331

[13] Ras M, Reitsma JB, Hoes AW, Six AJ, Poldervaart JM (2017). Secondary analysis of frequency, circumstances and consequences of calculation errors of the HEART (history, ECG, age, risk factors and troponin) score at the emergency departments of nine hospitals in the Netherlands. BMJ Open.

[14] Chen XH, Jiang HL, Li YM, Chan CPY, Mo JR, Tian CW, Lin PY, Graham CA, Rainer TH (2016). Prognostic values of 4 risk scores in Chinese patients with chest pain: prospective 2-Centre cohort study. Medicine (Baltimore).

[15] Sun BC, Laurie A, Fu R, Ferencik M, Shapiro M, Lindsell CJ, Diercks D, Hoekstra JW, Hollander JE, Kirk JD, Peacock WF, Anantharaman V, Pollack CV (2016). Comparison of the HEART and TIMI risk scores for suspected acute coronary syndrome in the emergency department. Crit Pathw Cardiol.

[16] Poldervaart JM, Langedijk M, Backus BE, Dekker IMC, Six AJ, Doevendans PA, Hoes AW, Reitsma JB (2017). Comparison of the GRACE, HEART and TIMI score to predict major adverse cardiac events in chest pain patients at the emergency department. Int J Cardiol.

[17] Laureano-Phillips J, Robinson RD, Aryal S, Blair S, Wilson D, Boyd K, Schrader CD, Zenarosa NR, Wang H (2019). HEART score risk stratification of low-risk chest pain patients in the emergency department: a systematic review and Meta-analysis. Ann Emerg Med.

[18] Al-Zaiti SS, Faramand Z, Alrawashdeh MO (2019). Comparison of clinical risk scores for triaging high-risk chest pain patients at the emergency department. Am J Emerg Med.

[19] Ong ME, Lee Ng CH, Goh K (2012). Prediction of cardiac arrest in critically ill patients presenting to the emergency department using a machine learning score incorporating heart rate variability compared with the modified early warning score. Crit Care.

[20] Jang DH, Kim J, Jo YH, Lee JH, Hwang JE, Park SM, Lee DK, Park I, Kim D, Chang H (2020). Developing neural network models for early detection of cardiac arrest in emergency department. Am J Emerg Med.

[21] Goto T, Camargo CA, Faridi MK, Freishtat RJ, Hasegawa K (2019). Machine learning-based prediction of clinical outcomes for children during emergency department triage. JAMA Netw Open.

[22] Raita Y, Goto T, Faridi MK, Brown DFM, Camargo CA, Hasegawa K (2019). Emergency department triage prediction of clinical outcomes using machine learning models. Crit Care.

[23] Aerts JM, Haddad WM, An G, Vodovotz Y (2014). From data patterns to mechanistic models in acute critical illness. J Crit Care.

[24] Expert consensus group on emergency pre-examination and triage. Expert consensus on emergency pre-examination and triage. Chin J Emerg Med. 2018;27(6):599–604.

[25] Editorial Committee of Chinese Journal of Cardiovascular Diseases EGoSEaDoCP., Expert Group on Standardized Evaluation and Diagnosis of Chest Pain (2014). Chinese Expert Consensus on Standardized Evaluation and Diagnosis of Chest Pain. Chin Circul J.

[26] Emergency Medicine Branch of Chinese Medical Association CPBoCHIEPA, Chest Pain Branch of China Healthcare International Exchange Promotion Association (2019). Consensus for emergency diagnosis and treatment of acute chest pain. Chin J Emerg Med.

[27] Jacobs I, Nadkarni V, Bahr J (2004). Cardiac arrest and cardiopulmonary resuscitation outcome reports: update and simplification of the Utstein templates for resuscitation registries: a statement for healthcare professionals from a task force of the International Liaison Committee on Resuscitation (American Heart Association, European Resuscitation Council, Australian Resuscitation Council, New Zealand Resuscitation Council, Heart and Stroke Foundation of Canada, InterAmerican Heart Foundation, Resuscitation Councils of Southern Africa). Circulation.

[28] Penttilä I, Penttilä K, Rantanen T (2000). Laboratory diagnosis of patients with acute chest pain. Clin Chem Lab Med.

[29] Obermeyer Z, Emanuel EJ (2016). Predicting the future - big data, machine learning, and clinical medicine. N Engl J Med.

[30] Liu N, Lee MA, Ho AF (2014). Risk stratification for prediction of adverse coronary events in emergency department chest pain patients with a machine learning score compared with the TIMI score. Int J Cardiol.

[31] Riley RF, Newby LK, Don CW, Roe MT, Holmes DJN, Gandhi SK, Kutcher MA, Herrington DM (2013). Diagnostic time course, treatment, and in-hospital outcomes for patients with ST-segment elevation myocardial infarction presenting with nondiagnostic initial electrocardiogram: a report from the American Heart Association Mission: lifeline program. Am Heart J.

[32] Sakamoto JT, Liu N, Koh ZX, Fung NXJ, Heldeweg MLA, Ng JCJ, Ong MEH (2016). Comparing HEART, TIMI, and GRACE scores for prediction of 30-day major adverse cardiac events in high acuity chest pain patients in the emergency department. Int J Cardiol.

[33] Liu N, Guo D, Koh ZX, Ho AFW, Xie F, Tagami T, Sakamoto JT, Pek PP, Chakraborty B, Lim SH, Tan JWC, Ong MEH (2020). Heart rate n-variability (HRnV) and its application to risk stratification of chest pain patients in the emergency department. BMC Cardiovasc Disord.

[34] Liu N, Goh J, Lin Z, Koh ZX, Fook-Chong S, Haaland B, Wai KL, Ting BP, Shahidah N, Ong MEH (2014). Validation of a risk scoring model for prediction of acute cardiac complications in chest pain patients presenting to the emergency department. Int J Cardiol.

[35] Sakamoto JT, Liu N, Koh ZX, Guo D, Heldeweg MLA, Ng JCJ, Ong MEH (2018). Integrating heart rate variability, vital signs, electrocardiogram, and troponin to triage chest pain patients in the ED. Am J Emerg Med.

